# Post-Intubation Tracheal Stenosis: Analysis of 250 Operated Cases and the Legacy of Professor Vicente Forte

**DOI:** 10.1590/0100-6991e-2026005625-en

**Published:** 2026-04-09

**Authors:** VICENTE FORTE, ANDRE MIOTTO, RODRIGO CAETANO DE SOUZA, MARIO CLAUDIO GHEFTER, PETRUCIO ABRANTES SARMENTO, MARIA ALENITA DE OLIVEIRA, JAQUELINA SONOE OTA ARAKAK, JOAO ALESSIO PERFEITOI

**Affiliations:** 1 - Escola Paulista de Medicina - UNIFESP (Disciplina de Cirurgia Torácica), São Paulo - São Paulo, Brasil; 2 - Hospital do Servidor Publico Estadual São Paulo - São Paulo, Brasil; 3 - Universidade Federal da Paraíba João Pessoa - Paraíba, Brasil; 4 - Escola Paulista de Medicina - UNIFESP São Paulo - São Paulo, Brasil

## INTRODUCTION

Vicente Forte (1937-2008) was born in São Paulo on March 15, 1937, in the Brás district, the son of Alfredo Forte and Rosina Marquetti Forte. He graduated from the Escola Paulista de Medicina (EPM) in 1961 (now part of the Universidade Federal de São Paulo) and later became a faculty member at EPM, working in Thoracic Surgery under his mentor and idol, Professor Costabile Gallucci. 

Throughout his academic career, he completed a master’s degree, a doctorate, and the Brazilian teaching habilitation. His curriculum was extensive, with numerous publications, and his presence as a teacher was always remarkable. Although he worked across several areas of Thoracic Surgery, tracheal surgery was his greatest passion. It is said that, while still a resident, he witnessed a young patient being told that nothing could be done at the time and that he would need to live permanently with a tracheostomy tube. This experience led him to pursue a career in tracheal surgery. That patient remained his lifelong friend and was later operated on and successfully decannulated by Professor Forte, who did not easily accept limitations and relished challenges. 

He visited several Thoracic Surgery centers abroad and trained with Professor Hermes Grillo, a recognized pioneer in tracheal surgery, with whom he developed a close friendship. Restless by nature, he did not rest until he led and performed the first lung transplant in the state of São Paulo on June 20, 1990, at Hospital São Paulo of the Escola Paulista de Medicina. 

In addition to his academic career, he maintained a busy clinical practice, was greatly admired by patients, and participated actively in medical societies. He became a specialist in Cardiovascular Surgery (1977) and Thoracic Surgery (1981); a founding member and first president of the Brazilian Society of Thoracic Surgery (SBCT); president of the South American Society of Thoracic Surgery (1998-1999); and an emeritus member of the Brazilian College of Surgeons. He participated in more than 600 national and international congresses. A voracious reader, tireless worker, and lifelong learner, he authored more than 70 book chapters. 

His Italian heritage was evident in his deep love for his family, especially for Lílian, his devoted wife and lifelong partner. He had two daughters and one son, a source of great pride for him, who, in turn, gave him several grandchildren. He loved being called “Nono” by his grandchildren and by many friends, who affectionately adopted the nickname as well. In his honor, “Nono Vicentinho” is now a comic-book character featured in SBCT News (the SBCT’s monthly bulletin). 

Vicente passed away prematurely while still highly active, at the age of 71, on April 24, 2008, due to a myocardial infarction. Alongside the many names he acknowledged in his habilitation thesis, he left words that reflect his personality and philosophy of life: “Unfortunate is the one who forgets those who decisively influenced his life, wasting the opportunity to honor them and keep them alive in some way. For me, there is no science without love. My entire life has been devoted to learning the art of Medicine with the brain, but also with the heart.” 

His example is immortal, and his legacy is enduring. The Thoracic Surgery Division of EPM continues to follow Professor Vicente Forte’s path, always citing him with honor and deep affection. His habilitation thesis, not yet published, describes the work of his life, demonstrating the excellence and vast experience he achieved. Here, we objectively present this highly significant body of work.

### The work of a lifetime

 The trachea is a flexible, compressible tube that extends through the neck and mediastinum. Narrowing of this tube may result in tracheal stenosis, which can be congenital or acquired^1^. Both endotracheal tubes, used for orotracheal or nasotracheal intubation, and tracheostomy cannulas are widely employed to provide mechanical ventilatory support in cases of respiratory failure. However, these devices may cause injury due to excessive cuff pressure^2^. 

Tracheobronchial stenosis is a serious condition of multifactorial etiology and has several treatment options. Since 1886, various approaches have been used for its management, including tracheal incision, exclusion procedures, tracheoplasty, resection with anastomosis, grafts, and flaps. Despite technical advances, there is still no absolute consensus regarding the optimal approach for managing postintubation tracheal stenosis. Interventional endoscopic procedures and surgical approaches represent the current treatment options, and new modalities continue to be evaluated for efficacy and safety^1^ ^,^ ^3^ ^,^ ^4^. 

Surgical resection with reconstruction is considered the gold standard for treating post-intubation tracheal stenosis. However, not all patients are ideal candidates for surgery, as this decision depends on stenosis characteristics and associated comorbidities^4^ ^-^ ^5^. 

Despite the inherent procedural risks, surgery is generally successful when patients are carefully selected. There is no consensus regarding the optimal timing of extubation after tracheal resection, and this decision is based on the patient’s clinical condition^6^ ^-^ ^7^. 

In this study, we present our accumulated experience in the resection of postintubation tracheal stenosis with tracheal reconstruction by tracheotracheal, cricotracheal, or laryngotracheal anastomosis.

## METHOD

Consecutive patients who underwent surgery between 1969 and 1996 for postintubation tracheal stenosis at a tertiary medical center were included. All details were recorded at the time of the procedures, followed by a detailed retrospective analysis. 

This manuscript is based on a historical cohort of patients operated on between 1969 and 1996, a period before the implementation of current Research Ethics Committee systems in Brazil. Given the retrospective nature of the material, the procedures performed at different institutions over several decades, and the impossibility of obtaining retrospective informed consent - since a substantial proportion of patients have died or cannot be located - submission of the study to an institutional ethics committee was not feasible. All data were analyzed in an aggregated, anonymized form, with no possibility of individual identification, in accordance with ethical principles of confidentiality, respect for human dignity, and nonmaleficence. 

All patients had confirmed tracheal or tracheobronchial stenosis. At least one imaging study was requested, including linear planigraphy of the cervicothoracic trachea, xeroplanigraphy of the cervicothoracic trachea, static and dynamic tracheography, cervicothoracic computed tomography, cervicothoracic magnetic resonance imaging, or esophagography. 

Imaging studies were used to evaluate stenosis characteristics, including location, number, length, and the presence or absence of laryngeal stenosis. Based on these data, the percentage of trachea to be resected was calculated, and stenosis was classified into three groups:

- Short: resection of up to 20% of the trachea;
- Intermediate: resection between 20% and 40%;
- Long: resection exceeding 40%.

All patients underwent laryngotracheoscopy to confirm the diagnosis and assess stenosis characteristics. In selected cases, spirometry with flow-volume curves and arterial blood gas analysis were also requested. Additional tests were ordered according to age, clinical history, or abnormalities found on physical examination. In all cases, the stenotic segment was resected by removing a cylindrical portion of the trachea, followed by immediate reconstruction using tracheotracheal, cricotracheal, or laryngotracheal anastomosis. 

The primary surgical approach was cervicotomy, with sternotomy or thoracotomy used as an alternative or combined approaches when necessary. Regardless of the approach, after exposure of the trachea, the stenotic area was identified and dissected using sharp and blunt techniques. Fibrotic tissues were divided close to the external surface of the trachea to retract the paratracheal tissues and the right and left inferior laryngeal nerves laterally. Tracheal rings were transected a few millimeters above and below the previously defined stenotic segment. 

The tracheal margins were examined to confirm normal appearance and consistency of the rings, as well as adequate bleeding and absence of ischemia. 

In patients with tracheostomy, the stoma was resected together with the stenosis (when located in the same segment), sutured (if distant from the lesion), or used as an exit site for the external limb of a Montgomery T-tube, as needed. Previously healed tracheostomies located away from the stenosis were not manipulated. 

In cases where proximity to suture lines posed a risk, the brachiocephalic trunk was secured with sutures below the anastomosis or separated from the trachea using pretracheal muscles or an expanded polytetrafluoroethylene conduit to prevent friction. 

Stenoses involving the larynx were always corrected simultaneously with tracheal reconstruction. Techniques varied according to the location and severity of laryngeal involvement and included anterior subglottic enlargement with tracheal or costal cartilage, posterior cricoid expansion, partial or extended cricoid resection, or a combination of these approaches. In cases requiring laryngeal enlargement with cartilage grafts, a Montgomery T-tube was maintained until neomucosal coverage of the graft had developed. 

After surgery, patients were admitted to the intensive care unit as a precautionary measure. They received continuous nebulization, respiratory therapy, analgesia, and antibiotics. In the absence of complications, patients resumed a regular diet and ambulation one day after surgery and were discharged from the ICU according to medical judgment. During the first three postoperative days, patients were closely monitored for upper airway obstruction or supra- or subglottic edema resulting from incomplete correction or inflammatory processes. 

Incisions were inspected daily for signs of anastomotic dehiscence or infection. Cervical, pleural, or mediastinal drains were removed after 48 hours if there was no significant drainage of blood, secretions, or air. 

Patients with a T-tube received instructions regarding hygiene, suctioning, and signs of obstruction and were advised to return immediately to the hospital if obstructive symptoms occurred. 

After hospital discharge, patients returned periodically for clinical follow-up. In-hospital complications were classified as mild, moderate, or severe according to clinical findings and were managed accordingly. 

Patients who underwent sternotomy or thoracotomy received chest radiographs to assess lung expansion. For those treated via a cervical approach, radiographs were requested only in the presence of complications. 

Surgical outcomes were evaluated based on both symptoms and laryngotracheoscopic findings, including the degree of inflammation, dehiscence, or residual stenosis

### Bronchoscopic classification of surgical outcomes:

- Excellent: perfect anastomosis (no symptoms);
- Good: anastomosis with up to 20% restenosis (asymptomatic);
- Fair: anastomosis with 20-40% restenosis (symptomatic);
- Poor: anastomosis with > 40% restenosis (symptomatic).

Demographic data, dates of operations, indication and duration of prior intubation, surgical approach, symptoms, previous treatments, and comorbidities were collected. 

Nominal categorical variables were compared between groups using the chisquare test or Fisher’s exact test. The level of statistical significance was set at p<0.005.

## RESULTS

Between March 1969 and February 1996, data were collected from 250 consecutive patients who underwent surgery for post-intubation tracheal stenosis. 

Of the included patients, 58.4% were male, with a mean age of 35.6 years (range: 6 months to 76 years). Children (≤12 years) accounted for 12.4% of the sample, and adults (> 2 years) for 87.6%. Regarding race, 206 patients were White, 40 were Black, and 4 were Asian. The most frequent indications for intubation were traumatic brain injury (35.2%) and postoperative complications following cardiac surgery (24.4%). 

The duration of intubation associated with stenosis ranged from a few hours (in elective surgical procedures) to 90 days, with a mean of 13.3 days and a median of 10 days. The most common durations were a few hours to 7 days (34.5%) and 7 to 14 days (31.0%).

Eighty patients underwent surgery with evidence of ventilatory failure (none of whom had a tracheostomy). Seventeen patients (6.8%) had a prior diagnosis of bronchial asthma. 

Linear planigraphy was performed in 203 patients (81.2%), and all 250 patients underwent laryngotracheoscopy. Based on linear planigraphy, the length of stenosis was measured, and the percentage of trachea resected was calculated, classifying stenosis as short (≤ 20%), intermediate (20-40%), or long (40-70%). This classification was useful in anticipating intraoperative challenges and preventing complications.

- 66.8% of patients had tracheal stenosis;
- 31.6% presented laryngotracheal stenosis (most commonly subglottic);
- 1.6% had tracheal stenosis associated with tracheoesophageal fistula.

Among cases of laryngotracheal stenosis, 25.6% were corrected during the same procedure, as were all cases involving tracheoesophageal fistula. Among patients with isolated tracheal stenosis, 68.4% were located in the proximal third of the trachea, 4.8% were double stenoses, and 3.6% represented restenosis following prior surgery performed at other institutions. 

Eighty-two patients (32.8%) had undergone previous treatments, primarily dilation of the stenosis and placement of a T-tube. Other prior interventions included tracheoplasty, laryngoplasty, laser surgery, microsurgery, arytenoidectomy, and excision of granulomas. 

Hypertrophic skin scarring was observed in 44.8% of patients, likely related to an abnormal connective tissue response with increased fibroblast proliferation. This complication occurred in 57.1% of Black and Asian patients and in 45.6% of White patients, with no statistically significant difference between groups. 

Most surgeries were performed via a cervical approach (94.4%). Stenosis length ranged from 0.5 to 8.0cm (mean and median: 3.5cm), most commonly between 2.0 and 5.0cm (70.8%). Intermediate length stenoses were the most frequent (36.6%). To reduce tension at the suture line, laryngeal release was performed in 9 patients. The internal diameter of the stenosis ranged from 2.0 to 8.0mm (median: 4.0mm). 

Among patients with laryngotracheal stenosis, partial cricoid resection was performed in 9.2%, extended resection in 6.4%, and anterior, posterior, or combined laryngeal expansion with tracheal or costal cartilage in 10.0%. 

Outcomes were similar between patients with isolated tracheal stenosis and those with laryngotracheal stenosis.

### Immediate Postoperative Complications

- No complications: 82.4%;
- Prolonged ICU stay (> 2 days) due to intubation: 5.2%;
- Surgical wound infection: 3.6%;
- Minor anastomotic dehiscence: 1.6%;
- Pneumothorax: 1.2%;
- Sternal osteomyelitis with mediastinitis: 0.8%;
- Cervical hematoma: 0.4%;
- Right upper lobe atelectasis: 0.4%.

### Mortality

- In-hospital mortality: 4% (n = 10);
- Pneumonia with sepsis: 3 cases;
- Severe cervical and mediastinal infection: 2 cases;
- Sudden death: 2 cases;
- Severe intraoperative arrhythmia: 1 case;
- Severe hemorrhage from previously operated larynx: 1 case;
- Respiratory failure due to advanced pulmonary emphysema: 1 case.

In late follow-up (>30 days), 82.1% of patients had no complications. There were 34 cases (13.6%) of anastomotic restenosis, 8 requiring maintenance of a T-tube, and 1 resulting in death. 

Follow-up included laryngotracheoscopy, performed at least once in all patients, approximately on postoperative day 14. Follow-up duration ranged from 1 month to 27 years (mean: 60.9 months; median: 43.5 months).

### When grouped

- 212 patients (84.8%) had excellent or good results;
- 20 patients (8.0%) had fair or poor results;
- 8 patients were not reevaluated;
- 10 patients died (4%).

Children had a lower incidence of associated laryngotracheal stenosis compared with adults, although surgical outcomes were similar. 

Stenosis following orotracheal intubation was more frequently located in the proximal third of the trachea, whereas post-tracheostomy stenosis was more common in the middle and distal thirds, without statistical significance. 

Outcomes were significantly better in primary surgeries than in reoperations for restenosis. No significant difference was observed among types of anastomosis (tracheotracheal, cricotracheal, or laryngotracheal) (χ² = 2.56), nor among short, intermediate, and long stenoses (χ² = 2.69). 

In patients who had not yet completed a four-month interval - during which the acute inflammatory process is gradually replaced by chronic inflammation - and who presented with dyspnea, tracheal dilation followed by T-tube placement was indicated.

## DISCUSSION

Many authors have traditionally believed that laryngotracheal stenosis occurs only in patients intubated for prolonged periods, characterizing so-called “prolonged intubation”^8^ ^,^ ^9^. In the ICUs we followed, the maximum duration of orotracheal or nasotracheal intubation ranged from 5 to 14 days. 

In the present study, the shortest duration of orotracheal intubation was 3 hours, and the longest was 90 days. Among the 226 patients intubated via the orotracheal route (the remaining 24 underwent primary tracheostomy), 4 remained intubated for less than 24 hours. Notably, 34.0% developed stenosis after intubation periods ranging from only a few hours to 7 days - an interval considered too short to attribute stenosis solely to intubation duration. 

Thus, the hypothesis that duration alone is responsible for stenosis is not supported. Studies have demonstrated that the severity of tracheal injury is a more decisive factor than duration, explaining cases such as the four patients in this series who developed stenosis after only a few hours of intubation^9^ ^-^ ^11^. 

In the larynx, compression by the external surface of the tube, and in the trachea, cuff pressure may compromise mucosal blood flow, leading to necrosis, edema, ulceration, and collapse. Prolonged compression of the cricoid cartilage^12^ ^-^ ^14^ or tracheal rings may result in cartilaginous necrosis, subsequently replaced by fibrous tissue. If necrosis progresses more rapidly than tissue repair, perforation of the tracheal wall may occur, resulting in posterior tracheoesophageal fistula or anterior tracheoarterial fistula^13^ ^-^ ^14^. 

High-volume cuffs can be inflated at lower pressures, thereby reducing injury. The introduction of high-volume, low-pressure cuffs significantly decreased the frequency of tracheal lesions. Materials such as silicone exert lower transtracheal pressure than PVC or latex^15^ ^-^ ^16^. 

In addition to mechanical pressure, other factors may aggravate tracheal injury^13^ ^,^ ^17^ ^-^ ^22^:

- Predisposing factors: age, sex, mucosal fragility, airway anatomy;
- Contributing factors: impaired wound healing, presence of a nasogastric tube, accumulation of contaminated secretions above the cuff, cervical surgery;
- Primary determinants: traumatic intubation, tube friction, tube material, excessive cuff pressure.

**Figure 1:**
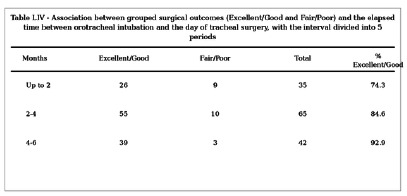
Figure showing the table of intervals between intubation and surgery, along with statistical analysis

The interval between the date of orotracheal intubation and the date of tracheal surgery is critically important for normal anastomotic healing. When the time between intubation and surgery was analyzed, subdivided into five intervals, overall and grouped surgical outcomes were significantly worse when patients were operated on within 4 months of intubation (Figure 1). 

The limitations of this study include its retrospective design and the fact that it was conducted over several decades in an earlier era, without prior publication. Nevertheless, the relevance of these data is unquestionable, as they represent one of the largest series reported worldwide, conducted by one of the foremost figures in tracheal surgery. 

The technique of tracheal resection and reconstruction has changed little over the past decades, and our results are comparable to those reported by leading international centers:

- Massachusetts General Hospital (Wright, 2004): overall complication rate 18.2% (ours: 17.2%)^23^ ^-^ ^25^;
- Cottingham, England (Sharpe et al., 1996): series of 82 cases, similar outcomes^26^;
- Brazilian experience (Bibas et al., 2014): 94 patients, equivalent complication rates^27^.

These findings demonstrate that Vicente Forte’s Brazilian experience achieved a technical standard comparable to that of the world’s leading reference centers.

## CONCLUSION

Resection of tracheal stenosis followed by reconstruction with tracheotracheal, cricotracheal, or laryngotracheal anastomosis proved highly effective for the treatment of mature stenoses (more than four months after intubation), with low morbidity and mortality and durable outcomes. 

Recurrence (restenosis) was more frequent among previously treated patients, those with hypertrophic skin scarring, and those operated on within four months of intubation.

### A LEGACY

Beyond serving as a tribute and acknowledgment, this article seeks to disseminate Professor Vicente Forte’s extensive experience in this field and to contribute to the historical record of Brazilian tracheal surgery. 

His scientific and technical legacy remains a benchmark in Brazilian and international Thoracic Surgery, continuing to encourage and inspire new generations of surgeons. 
